# Antihypertensive and Vasorelaxant Effects of Citric Acid and Lemon Juice in Spontaneously Hypertensive Rats: In Vivo and Ex Vivo Studies

**DOI:** 10.3390/nu15173849

**Published:** 2023-09-03

**Authors:** Kozo Nakamura, Yumiko Suzuki, Kazuma Goto, Shohei Yamaguchi, Masanori Hiramitsu

**Affiliations:** 1 Department of Science and Technology, Graduate School of Medicine, Science and Technology, Shinshu University, 8304, Minamiminowa, Nagano 399-4598, Japan; 23as208k@shinshu-u.ac.jp (Y.S.); 22as204c@shinshu-u.ac.jp (K.G.); 2 Department of Bioscience and Biotechnology, Faculty of Agriculture, Shinshu University, 8304, Minamiminowa, Nagano 399-4598, Japan; syamaguchi@shinshu-u.ac.jp; 3 Institute of Agriculture, Academic Assembly, Shinshu University, 8304, Minamiminowa, Nagano 399-4598, Japan; 4 Pokka Sapporo Food and Beverage Ltd., 10, Okatome, Yaizu, Shizuoka 425-0013, Japan; Masanori.Hiramitsu@pokkasapporo-fb.co.jp

**Keywords:** hypertension, nutraceutical, diet, citric acid, lemon, vasorelaxant, flavonoid

## Abstract

Hypertension is a key risk factor for heart, brain, and kidney disease development. Fruit consumption has been associated with a decrease in blood pressure. Lemon juice, which contains antihypertensive compounds, may exert antihypertensive effects. However, no research has verified the antihypertensive effects of citric acid, the most abundant ingredient in lemon juice. In the present study, we demonstrated the antihypertensive effects of citric acid and lemon juice by performing single oral administration tests and the aortic ring assay using spontaneously hypertensive rats (SHRs). Single oral doses of both agents markedly reduced the systolic and diastolic blood pressures in the SHRs. In addition, both these agents relaxed the thoracic aorta from the SHRs; however, these effects were notably attenuated by the removal of the aortic endothelium. Orally administered citric acid was rapidly absorbed and metabolized in vivo. Among the functional compounds in lemon juice, citric acid was identified as the primary antihypertensive component. Although more detailed studies are required to validate our findings, the novel functional attributes of citric acid can achieve the normalization of blood pressure when it is consumed via diet.

## 1. Introduction

Hypertension is a critical health issue that notably increases the chances of heart-related and various other ailments. Cardiovascular diseases claim more lives each year than any other health complication. Globally, around 1.4 billion individuals suffer from high blood pressure, yet a mere 14% have managed to maintain it at a safe level, according to a 2019 survey. The World Health Organization (WHO) offers scientifically supported recommendations for treating adult hypertension. Given that the vast majority of deaths linked to heart ailments and strokes occur in low- to medium-income countries, the prevention of hypertension should be a major focus area in healthcare [1]. Hypertension has two main types: primary and secondary. Secondary hypertension, found in a limited number of patients with high blood pressure, has clear and specific causes. On the other hand, primary hypertension, which accounts for most cases of high blood pressure, is caused by complex interactions between the genetic background, many environmental factors, and the aging process [2]. An important environmental factor affecting blood pressure is lifestyle, including the daily diet [2]. The “Dietary Approaches to Stop Hypertension (DASH)” diet, recommended by The National Institutes of Health, USA, is among the most important and well-established list of effective lifestyle interventions [2]. The DASH diet emphasizes an increased consumption of fruits, vegetables, and fat-free or low-fat dairy products, as well as a reduced consumption of sugar and fat [3]. Epidemiological studies have also shown that increased fruit consumption is associated with decreased blood pressure in adults [4]. In recent years, research focusing on improving blood pressure through dietary modifications, including antihypertensive nutraceuticals and functional foods, has gained significant attention from researchers [5].

Incorporating citrus fruits into the daily diet could potentially improve blood pressure. It might also be essential in the DASH diet [6,7]. Lemons and limes, distinct fruits often grouped in production statistics because of their similar uses, are the third most-produced citrus fruits globally, with 20,828,739 tons being produced in 2021 [8]. Because of its low sugar content, lemon is mainly used as a source of juice for seasoning or a beverage ingredient rather than being consumed as a fresh fruit. 

Several reports have demonstrated a relationship between lemon juice and blood pressure. Among Turkish patients with hypertension using alternative therapies, 40% drank lemon juice to ameliorate their higher blood pressure [9]. In addition, a significant negative correlation was observed between lemon consumption and systolic blood pressure (SBP) in healthy Japanese women [10]. Feeding spontaneously hypertensive rats (SHRs) diluted lemon juice and libitum for 90 days tended to suppress their blood pressure [11]. However, such studies did not confirm the antihypertensive effects of lemon juice.

The major nutrients of lemon juice include carbohydrates (8.6/100 g fresh weight [F.W.]) and organic acids (6.7/100 g F.W.), with the most abundant organic acid being citric acid (6.5/100 g F.W.) according to the Food Composition Database [12], which is based on the Standard Tables of Food Composition in Japan 2020 [13]. Citric acid plays a central role in the citric acid cycle to generate energy in all aerobic organisms; in addition, it has antioxidant properties that reduce the risk of chronic diseases such as heart disease and cancer through neutralization of harmful free radicals and help prevent the formation of kidney stone through chelation of calcium (Ca) ions [14,15]. However, we could not find any reports on the antihypertensive effects of citric acid.

Potassium citrate has been reported to lower blood pressure in patients with hypertension, and potassium (K) has been identified as the active component for lowering blood pressure in the literature [16,17]. Lemon juice is also characterized by higher K (100 mg/100 g F.W.) and vitamin C (50 mg/100 g F.W.) contents than other general foods [18]. K is widely recognized as a hypotensive mineral [19,20] and a major antihypertensive component of the DASH diet [21,22]. Taking 590 mg/day of K reduces blood pressure by 1 mmHg [23]. Recently, flavonoids and γ-aminobutyric acid (GABA) in lemon juice have been reported to exhibit antihypertensive effects [18,24]. The major flavonoids in lemon are eriocitrin and hesperidin, whose contents in lemon juice are estimated to be 4.07 mg/100 g F.W. and 2.27 mg/100 g F.W., respectively [11], and the GABA content is reported to be 85−320 mg/100 g F.W. [24].

The antihypertensive effect of vitamin C has been reported; an average daily intake of 500 mg has been shown to improve blood pressure in adults significantly [25]. Twelve weeks of sustained intake of 345 mg/day of hesperidin decreased the SBP of individuals with pre- and stage-1 hypertension [26]. A daily intake of 10 mg GABA may also improve blood pressure in adults [27]. In animal experiments using SHRs, *Lactobacillus*-fermented lemon juice, which has a high GABA content, has shown antihypertensive effects [24]. Therefore, we hypothesize that lemon juice, which contains antihypertensive compounds, exerts antihypertensive effects. 

In the present study, we aimed to evaluate the antihypertensive effects of citric acid and lemon juice using single oral administration tests and aortic ring assays in SHRs, which are commonly used animal models of hypertension. To confirm its antihypertensive effects, acid-flavonoid-reduced-lemon (AFR) juice was prepared, and its effects on SHRs were tested. We also discussed the antihypertensive components of lemon juice. To our knowledge, our study is the first to report the antihypertensive effects of citric acid and lemon juice. 

## 2. Materials and Methods

### 2.1. Reagents and Materials

Citric acid was purchased from Fuso Chemical Co., Ltd. (Osaka, Japan). Hydrochloric acid standard solution (1.0 mol/L), phenylephrine, acetylcholine, papaverine, isoflurane, and chemicals used to prepare Krebs solution were purchased from Kanto Chemical Co., Inc. (Tokyo, Japan). Mixed gas (95% O_2_, 5% CO_2_) was obtained from Okayasanso Co., Ltd. (Okaya, Japan), and physiological saline solution was obtained from Hikari Pharmaceutical Co., Ltd. (Tokyo, Japan). The Krebs solution was prepared at Shinshu University, and it was composed of 118 mmol/L NaCl, 4.7 mmol/L KCl, 2.5 mmol/L CaCl_2_, 1.2 mmol/L MgSO_4_, 1.2 mmol/L KH_2_PO_4_, 25 mmol/L NaHCO_3_, and 11.1 mmol/L glucose. Ultrapure water with a specific resistance of 18.2 MΩ × cm was prepared using an Arium 611 ultrapure water system (Göttingen, Germany).

AFR-lemon juice was provided by Sapporo Holdings Ltd. (Tokyo, Japan). The concentrations (mg/100 g) of citric acid, K, eriocitrin, hesperidin, and GABA in the lemon-derived samples are summarized in Table 1. The concentrations, excluding those of K and vitamin C, were defined based on the analytical results of the compositions using the external standard method with High-Performance Liquid Chromatography (HPLC) (SHIMADZU LC-20A) at Sapporo Holdings Ltd. The analytical conditions for each component were as follows. Citric acid: column Shodex RSPak KC-811, eluent A (0.2 mmol/L Bromothymol blue, 15 mmol/L Na_2_HPO_4_), eluent B (3 mmol/L perchloric acid), mobile phase A:B = 58.8:41.2 (isocratic), column temperature 50 °C, detection at 445 nm. Eriocitrin and hesperidin: column InertSustain C18 (5 μm, 4.6 × 250 mm, GL Science, Tokyo, Japan), eluent A (5% acetic acid), eluent B (methanol), mobile phase A:B = 70:30 (isocratic), column temperature 40 °C, detection wavelength 280 nm. GABA (AccQ, Tag derivative): column AccQ-Tag (3.9 × 150 mm, Waters, Milford, MA, USA), eluent A (sodium acetate, 30% trimethylamine, phosphoric acid), eluent B (60% acetonitrile), gradient min (B%) = 0 (0), 0.5 (2), 15 (7), 19 (10), 32 (33), 33 (33), 34 (100), column temperature 37 °C, fluorescence detection Ex250 nm EM395 nm. The K and vitamin C contents of lemon juice were determined at the Japan Food Research Laboratories (Tokyo, Japan) using conventional atomic absorption spectrometry and HPLC methods, respectively.

### 2.2. Animals and Ethics Statement

Spontaneously hypertensive male rats (SHRs/NCrlCrlj, Charles River Laboratories Japan, Inc., Kanagawa, Japan) were used for the animal studies. The rats were housed in plastic gauges with a room temperature of 23 ± 4 °C, humidity of 50 ± 20%, and a 12-h light-dark cycle (light period 7:00 am to 7:00 pm). During the rearing period, feed (MF; Oriental Yeast Co., Ltd., Itabashi, Japan) and tap water were provided ad libitum. Male 14–16-week-old SHRs and male 13–14-week-old SHRs were used for the aortic assay and single oral dose study, respectively. Male 23-week-old SHRs with available blood volumes were used for the hemodynamic studies. All animal studies were conducted in accordance with the animal experimental guidelines of the Faculty of Agriculture, Shinshu University, and were approved by the Animal Care Committee of the Faculty of Shinshu University (Approval No. 290061).

### 2.3. Single Oral Administration Test

#### 2.3.1. Administration Test for Citric Acid and Lemon Juice 

Thirty male 13- or 14-week-old SHRs were divided into five groups: the low-dose lemon juice group (*n* = 6, average body weight 332.1 ± 2.9 g, 10 mg/kg citric acid equivalent), high-dose lemon juice group (*n* = 6, average body weight 342.5 ± 6.9 g, 100 mg/kg citric acid equivalent), low-dose citric acid group (*n* = 6, average body weight 337.8 ± 5.7 g, 10 mg/kg citric acid), high-dose citric acid group (*n* = 6, average body weight 330.6 ± 1.9 g, 100 mg/kg citric acid), and the control group (*n* = 6, average body weight 337.4 ± 2.7 g). There were no significant differences in mean weight among the groups. The rats were fasted overnight and administered a single oral dose of citric acid or lemon juice solution (2.0 mL/kg) containing the respective doses of citric acid using a gastric sonde (Natsume Seisakusho Co., Ltd., Bunkyo, Tokyo, Japan). An equal volume of pure water was administered to the control group. The SBP and diastolic blood pressure (DBP) were measured using the tail-cuff method with a blood pressure monitor (BP-98A; Softron Co., Tokyo, Japan) at 0, 3, 6, 9, and 24 h after administration.

#### 2.3.2. Administration Test for AFR-Lemon Juice 

Eight 13- or 14-week-old male SHRs were divided into an AFR-lemon juice group *(n* = 4) and a control group (*n* = 6). Each group received AFR-lemon juice and pure water at 2.0 mL/kg, the same amount as Section 2.3.1. The single oral administration to each group and blood pressure measurements were performed as in Section 2.3.1.

#### 2.3.3. Citric Acid Hemodynamics in SHRs

The SHRs with surgically inserted portal vein catheters [28] were divided into two groups: citric acid group (*n* = 3) and control group (pure water, *n* = 3), each receiving 90 mg/kg (0.47 mmol/kg) of citric acid and pure water, respectively. Portal venous blood (200 µL) was collected via a catheter before (0 h) and at 0.25, 0.5, 1, 3, 6, 9, and 24 h after administration, mixed with 10 µL of 75 mmol/L EDTA-2Na solution, and centrifuged (1000× *g*, 10 min) to obtain plasma. Samples were prepared from plasma according to the following procedure. First, 0.53 mol/L HClO_4_ solution was added to 20 µL of plasma (to achieve a final concentration of 0.40 mol/L), mixed vigorously with a vortex mixer (1.0 min), and then centrifuged (1336× *g*, 3 min) to remove the proteins; the deproteinized supernatant was collected. Next, 750 µL of the analytical solvent was added to 50 µL of the supernatant; this mixture was diluted 16-fold to obtain an analysis sample.

The citric acid concentration in the blood samples was determined using an external standard method using a liquid chromatography-tandem mass spectrometry (LC-MS/MS) system (ultra-performance LC: Nexera-i and MS: LCMS–8045, Shimadzu Co., Kyoto, Japan). The LC-MS/MS analysis conditions were as follows: mobile phase, 0.1% water with formic acid; YMC Triart-PFP column (4.6 × 250 mm, 5 µm); flow rate, 0.50 mL/min; column temperature, 40 °C; injection volume, 5 µL; ESI ionization method (-); MRM analysis mode; and MRM transition m/z 191.00 [M − H] → m/z 110.95 (citric acid). 

Calibration curve preparation and LC-MS/MS quantification were performed as follows. Deproteinized supernatants obtained from untreated SHRs were diluted 4-fold in an analytical solvent used as a blank. These solutions were mixed 1:1 with 250, 500, 1000, and 2000 ng/mL citric acid standard solutions and with an analytical solvent to prepare 125, 250, 500, and 1000 ng/mL citric acid-deproteinized supernatant samples, respectively. LC-MS/MS analysis was performed, and the area of the blank sample was subtracted from the area of the citric acid-deproteinized supernatant samples to construct a calibration curve. The area value of the sample obtained by LC-MS/MS analysis was substituted into the calibration curve equation to calculate the citric acid concentration and multiplied by the dilution factor to get the citric acid concentration in the blood.

### 2.4. Aorta Assay

Aorta assays were performed as described previously [29]. After being anesthetized using isoflurane, the SHRs were subjected to laparotomy and exsanguination, and the thoracic aorta was excised rapidly. The excised aorta was immersed in physiological saline solution to wash away the blood, and the connective and adipose tissues adhering to the blood vessels were removed in Krebs solution, creating endothelium-intact aortic ring specimens with widths of approximately 2−3 mm. Endothelium-removed aortic ring specimens were prepared by rubbing the endothelium of the preserved vascular rings against a filter paper to remove the endothelium. The ring specimens were attached to tension-measuring hooks in an organ bath of an isometric tension testing apparatus filled with Krebs solution (37 °C) aerated with a mixed gas (95% O_2_, 5% CO_2_). An optimal resting tension of 1.5 g was applied, and the specimens were equilibrated for 60 min, with fresh Krebs solution being exchanged every 15 min. 

After confirming the contractile response to phenylephrine (0.3 μmol/L, vasoconstrictor), acetylcholine (0.1 mmol/L, endothelium-dependent vasodilator) was added to verify the intactness or removal of the vascular endothelium. The ring specimens were washed three times with fresh Krebs solution, returned to the resting tension, and replaced with fresh Krebs solution. Phenylephrine (0.3 μmol/L) or KCl (60 mmol/L) was added to induce vasoconstriction when tension reached a stable state. Once the tension stabilized, the test samples were added cumulatively, and the change in vascular tension was measured using a UFER UM-203 isometric transducer (Iwashiya Kishimoto Medical Instruments Co., Ltd., Kyoto, Japan) with a UFER UC-5A Magnus system (Iwashiya Kishimoto Medical Instruments Co., Ltd.). The data were recorded and analyzed using a PowerLab data-acquisition device and LabChart Pro v8.1.13 software (ADInstruments Pty. Ltd., New South Wales, Australia). 

To investigate the vasorelaxant effects of citric acid and lemon juice on phenylephrine-contracted and KCl-contracted SHR thoracic aortae with and without endothelium, citric acid and lemon juice solutions were cumulatively added to the organ bath to achieve citric acid concentrations of 0.10, 0.30, 0.50, 1.0, 3.0, and 5.0 mmol/L in the chamber. To investigate the effect of pH on the vasorelaxation of citric acid and lemon juice, lemon juice and citric acid aqueous solution were added to the chamber to achieve a citric acid concentration of 5.0 mmol/L. The pH was measured using a pH meter (IM22P, DKK-Toa Co., Shinjuku, Tokyo, Japan), and 1.0 mol/L HCl solution was added to the chamber to adjust the pH in the citric acid- or lemon juice-containing chamber. To investigate the vasorelaxant effects of lemon juice, AFR-lemon juice was cumulatively added at a volume similar to that of lemon juice. Each experiment was repeated six times. The change in tension from maximum contraction was recorded as %vasorelaxation, and the 50% relaxant concentration (EC_50_) was determined from the relaxation curve.

### 2.5. Statistical Analysis

Results are presented as the mean ± standard error (S.E.). Citric acid concentrations in the blood were compared using the student’s *t*-test. Multiple comparisons of weight in the groups were performed using the Tukey-Kramer test after a one-way Analysis of Variance (ANOVA). Multiple comparisons of changes in vascular tension and blood pressure were performed using the Tukey-Kramer and Dunnett’s tests, respectively, after one-way ANOVA. *p* < 0.05 was considered to indicate statistically significant results. Analyses were performed using Microsoft Excel 365 (16.0.13901.20276; Microsoft Corp., Redmond, WA, USA). 

## 3. Results

### 3.1. Antihypertensive Effects of Citric Acid and Lemon Juice

#### 3.1.1. Blood Pressure-Lowering Effects of a Single Oral Administration of Citric Acid and Lemon Juice

**Figure 1:**
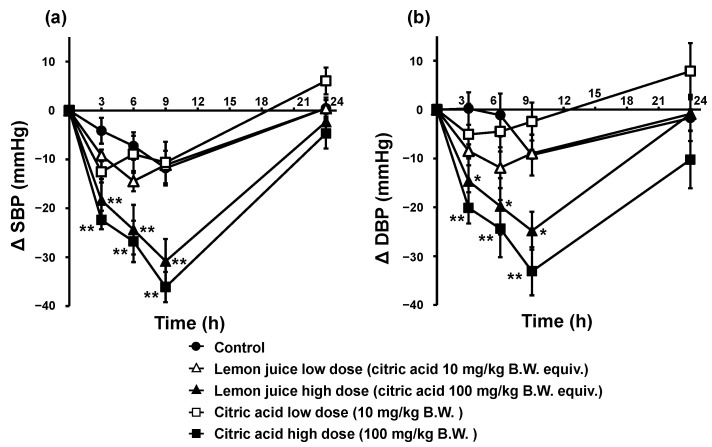
SBP (**a**) and DBP (**b**) trends in SHRs after a single oral administration of citric acid and lemon juice. Each plot represents the mean ± S.E. (*n* = 6); * *p* < 0.05, ** *p* < 0.01, compared with control; the indicated *p* values were obtained from the Dunnett’s tests. SBP: systolic blood pressure, DBP: diastolic blood pressure; SHR: spontaneous hypertensive rat, B.W.: body weight.

Changes in SBP and DBP in the single oral administration tests of citric acid and lemon juice in SHRs are shown in Figure 1. The changes in SBP and DBP were significantly lower in the high-dose citric acid and lemon juice groups than in the control group. The high lemon juice dose reduced SBP and DBP by −18.4 and −14.7 mmHg at 3 h, by −24.4 and −19.8 mmHg, respectively, at 6 h, and by −30.8 and −24.8 mmHg, respectively, at 9 h, with significant differences at all measurement points (*p* < 0.01 for SBP and *p* < 0.05 for DBP). High citric acid doses reduced SBP and DBP by −22.4 and −20.1 mmHg, respectively, at 3 h, by −26.8 and −24.4 mmHg, respectively, at 6 h, and by −36.1 and −33.1 mmHg, respectively, at 9 h, with significant differences at all measurement points (*p* < 0.01 for SBP and DBP). The kinetics of SBP and DBP in the high-dose citric acid and lemon juice groups were similar, with the lowest values at 9 h after administration and no significant differences at any measurement point (SBP: 0.361 < *p* < 0.724, DBP: 0.147 < *p* < 0.589). In contrast, in the low-dose citric acid and lemon juice groups, neither SBP nor DBP were significantly lower than those of the control (SBP: 0.113 < *p* < 0.909, DBP: 0.129 < *p* < 0.611).

#### 3.1.2. Citric Acid Hemodynamics

**Figure 2:**
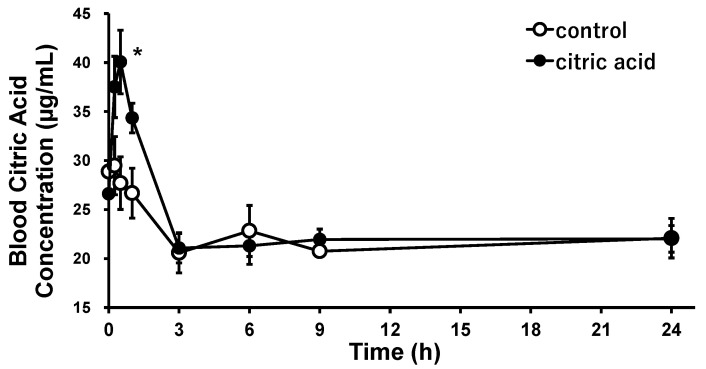
Citric acid concentration trends in the blood after a single oral administration of citric acid to the SHRs. Each plot represents the mean ± S.E. (*n* = 3); * *p* < 0.05, compared with control; the indicated *p* values were obtained from the Student’s *t*-test. SHR: spontaneous hypertensive rat.

The changes in blood citric acid concentrations over time after a single oral administration of citric acid to SHRs were investigated to evaluate the pharmacological bioavailability of citric acid. The results are shown in Figure 2. Blood citric acid concentration increased within a short period after oral administration, with a maximum concentration of 40.1 μg/mL (209 µmol/L) at 30 min (*p* < 0.05). Thereafter, the blood citric acid concentration declined rapidly, reaching almost the same concentration as before at 3 h after administration. The increases in citric acid concentration were significantly higher than those in the control group from 15 min to 1 h after administration, i.e., 10.9 µg/mL (56.8 µmol/L) at 15 min (*p* = 0.048), 13.5 µg/mL (70.0 µmol/L) at 30 min (*p* = 0.017), and 7.7 µg/mL (40.3 µmol/L) at 1 h (*p* = 0.0076). The average blood citric acid concentration in the control and the citric acid groups from 3 to 24 h did not change significantly at 21.6 µg/mL (112 µmol/L).

### 3.2. Vascular Effects of Citric Acid and Lemon Juice

#### 3.2.1. Vasorelaxation Effects of Citric Acid and Lemon Juice in Endothelium-Intact and -Removed Aorta

**Figure 3:**
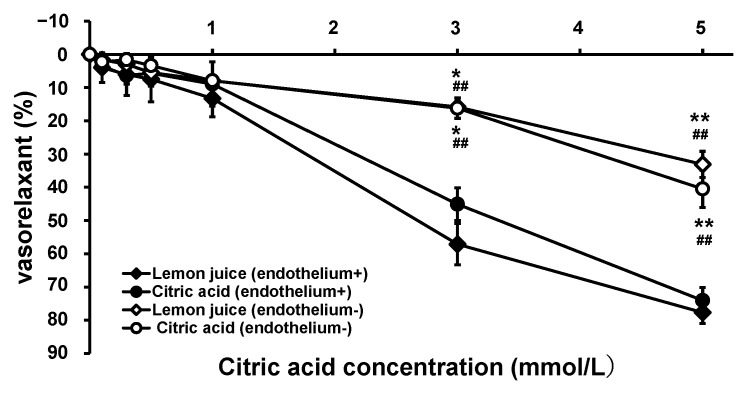
Concentration–vasorelaxation curves of thoracic aorta from SHRs after treatment with citric acid and lemon juice. Each plot represents the mean ± S.E. (*n* = 6); * *p* < 0.05, ** *p* < 0.01 vs. treatment with citric acid in the endothelium-intact aorta, ## *p* < 0.01 vs. treatment with lemon juice in the endothelium-intact aorta; the indicated *p* values were obtained from the Tukey-Kramer tests; SHR: spontaneous hypertensive rat.

To examine the antihypertensive mechanism and site of action of citric acid and lemon juice, the vascular action of the thoracic aorta from SHRs was measured after citric acid and lemon juice administration. Endothelium-intact (normal) and endothelium-removed SHR aortae were used for the tests. Figure 3 illustrates the changes in vascular tension following the treatments. In the endothelium-intact aorta, after citric acid and lemon juice treatments, weak dilations of 3.54% and 3.96%, respectively, began from an intra-chamber citric acid concentration of 0.10 mmol/L and strong vasodilation levels of 45.1% and 57.1%, respectively, were observed at 3.0 mmol/L, and of 74.1% and 77.7%, respectively, were observed at 5.0 mmol/L citric acid concentrations. The EC_50_ values were 3.68 mmol/L for citric acid and 2.67 mmol/L for lemon juice. Although greater relaxation was observed with lemon juice addition, there was no significant difference in vasodilation kinetics between the two treatments (0.160 < *p* < 0.971). The relaxing effects with citric acid and lemon juice solution were greatly attenuated, with both showing significant reductions at citric acid concentrations of 3.0 and 5.0 mmol/L in the SHR aortae subjected to endothelium removal compared with those in the endothelium-intact aortae.

#### 3.2.2. Effect of pH on Vasorelaxation

The pH of the Krebs solution in the aortic assay system aerated with a mixture of 95% O_2_ and 5% CO_2_ gas was 7.43. The pH change in the aortic assay system caused by the addition of citric acid and lemon juice may cause a vascular response owing to the changes in intracellular and extracellular conditions [30]. Therefore, to verify the pH-dependent vascular response after citric acid and lemon juice treatments, the vasorelaxant effects of lemon juice, citric acid, and hydrochloric acid were measured at the same pH. These results are summarized in Table 2. The pH in the chambers with citric acid and lemon juice at a citric acid concentration of 5.00 mmol/L were 6.08 and 6.53, respectively, and the corresponding chamber HCl concentrations for the same pH were 8.43 × 10^−4^ mmol/L and 3.01 × 10^−4^ mmol/L, respectively. As shown in Figure 3, at an intra-chamber citric acid concentration of 5.0 mmol/L, 74.1% and 77.7% vasorelaxation was caused by adding citric acid and lemon juice, respectively. In contrast, HCl at the same pH induced no vasorelaxation but vasoconstriction.

#### 3.2.3. Vasorelaxation with Citric Acid and Lemon Juice in KCl-Contracted Aortae from SHRs

**Figure 4:**
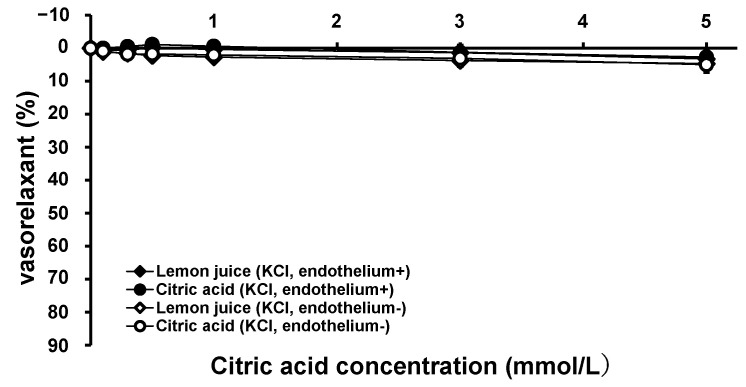
Concentration–vasorelaxation curves of KCl-contracted thoracic aorta from SHRs by treatment with citric acid and lemon juice in endothelium-intact and endothelium-removed aortae. Each plot represents the mean ± S.E. (*n* = 6); SHR: spontaneous hypertensive rat, KCl: potassium chloride.

To examine the vasorelaxation mechanism in greater detail, the vascular effects of citric acid and lemon juice in the KCl-constricted thoracic aortae from the SHRs were measured. Figure 4 shows the vascular tension trends with the cumulative addition of citric acid and lemon juice to the KCl-constricted SHR aortae with intact and removed endothelium. Citric acid and lemon juice solution had minimal vasorelaxation effects on KCl-constricted endothelium-intact and endothelium-removed vessels. There was no significant difference in vasorelaxation between the citric acid and lemon juice treatments in the endothelium-intact and endothelium-removed aortae at all measurement points (the endothelium-intact aortae: 0.507 < *p* < 0.967, the endothelium-removed aortae: 0.208 < *p* < 0.523).

### 3.3. Single Oral Administration Tests and Aorta Assay for Testing the Effects of AFR-Lemon Juice

**Figure 5:**
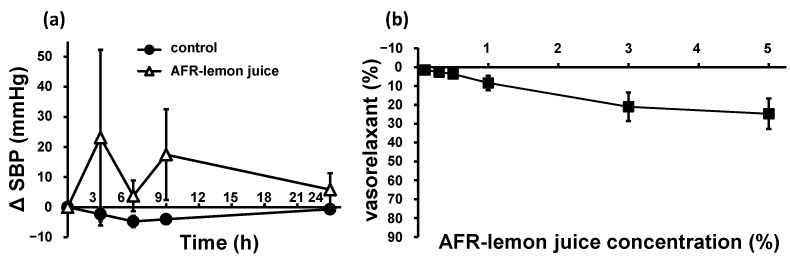
SBP trends (**a**) in SHRs after a single oral administration of AFR-lemon juice or pure water as control (control: *n* = 6, AFR-lemon juice: *n* = 4), and concentration–vasorelaxation curves (**b**) of endothelium-intact thoracic aorta from SHRs after treatment with AFR-lemon juice (*n* = 3). “AFR-lemon juice concentration (%)” in (**b**) mean the citric acid concentration when adding the same amount of lemon juice as the added AFR-lemon juice. Each plot represents the mean ± S.E.; AFR: acid-flavonoid-reduced; SBP: systolic blood pressure; SHR: spontaneous hypertensive rat.

The antihypertensive effects of AFR-lemon juice on the SHRs were tested and compared with those of pure water (control). The contents of citric acid, K, vitamin C, and the two flavonoid types in AFR-lemon juice were 14%, 71%, 50%, and 1.7% of those in lemon juice, respectively, whereas the GABA contents in the AFR-lemon juice and lemon juice was almost the same (see Table 1). The SBP trends are shown in Figure 5a. AFR-lemon juice did not decrease the SBP after administration but tended to increase blood pressure, but not significantly. The vasorelaxant effects of AFR-lemon juice were tested in SHR-derived endothelium-intact thoracic aortae after cumulative addition. The volume of AFR-lemon juice added was the same as that of lemon juice. The results are shown in Figure 5b. AFR-lemon juice exhibited a non-significant weak vasorelaxant effect.

## 4. Discussion

In the present single oral administration study on SHRs, significant decreases in both SBP and DBP were observed in the high-dose citric acid, and high-dose lemon juice groups from 3 h to 9 h, compared with those of the control group, and the antihypertensive effects of citric acid and lemon juice were demonstrated. To our knowledge, this is the first study to demonstrate the antihypertensive effects of orally administered citric acid and lemon juice on SHRs. This novel function of citric acid, a common food ingredient, can be used to facilitate health improvements through dietary intake. A dose of 100 mg/kg citric acid significantly reduced the SBP and DBP, whereas a dose of 10 mg/kg did not. The dose of citric acid required to reduce blood pressure in SHRs is estimated to be 10−100 mg/kg. The dose of citric acid administered to the SHRs weighing 300 g was calculated to be 30 mg, and the equivalent dose for an individual weighing 60 kg according to the Kleiber rule is assumed to be 1.60 g [31], corresponding to 35.4 g of lemon juice used in the present study. Individuals with higher blood pressure may benefit more from citric acid. The effects of food ingredients with antihypertensive properties vary with blood pressure, with considerable reports showing increased effectiveness at higher blood pressure [21,29,32,33]. The present study evaluated the antihypertensive and vasorelaxant effects of citric acid using SHRs. Therefore, the effects may be weak under normotensive conditions; this could be one of the reasons why the antihypertensive effects of citric acid, a common ingredient, have not yet been reported.

In addition to citric acid (4.5 g/100 g), the lemon juice used in the present study contained K (164 mg/100 g), vitamin C (38 mg/100 g), eriocitrin (2.2 mg/100 g), hesperidin (1.3 mg/100 g), and GABA (9.8 mg/100 mg), which are considered antihypertensive compounds. A lowering of blood pressure by 1 mmHg is expected with an intake of 590 mg/day of K [23]. The effective dose equals 360 g of lemon juice, which seems too high to consume daily. Since the effective daily vitamin C consumption dose is 500 mg [25], its content in lemon juice is too low to elicit an antihypertensive effect. Hayakawa et al. reported that 0.50 mg/kg GABA significantly reduced the SBP in SHRs after a single oral administration [34]. In the present study, GABA was present in lemon juice at 9.8 mg/100 g, and the GABA dose was calculated to be 0.22 mg/kg in the high-dose lemon juice group, which was below the effective dose. In addition, a single oral dose of AFR-lemon juice, which contained the same levels of GABA and less citric acid and flavonoids than lemon juice, did not reduce blood pressure in the SHRs. Therefore, the antihypertensive effects of GABA in lemon juice are limited. Flavonoids are key antihypertensive components of citrus fruits. Hesperidin is a major antihypertensive flavonoid in citrus fruits, including lemons [18]. In a study involving the single oral administration of 10 mg/kg hesperidin to SHRs, the SBP decreased significantly (−6.2 mmHg) at 12 h [32]. The hesperidin concentration in the lemon juice used in the present study was 1.3 mg/100 g, and the hesperidin intake in the high-dose lemon juice group was 0.029 mg/kg, which is 1/345 of the effective dose in SHRs [32]. The effective dose of eriocitrin, unique to lemons and rarely found in other citrus fruits [11], could not be verified because of the lack of reports on its effects on blood pressure in SHRs. The lemon flavonoid extract consisting of 62.4% eriocitrin and 16.5% hesperidin significantly suppressed the rise in blood pressure in SHRs from 4 to 13 weeks [11]. The daily effective dose of the extract was 7.72 mg, and that of eriocitrin and hesperidin was 4.82 mg and 1.27 mg, respectively. Eriocitrin and hesperidin intake in the present study was 0.017 mg and 0.010 mg, respectively, which is 1/588 and 1/1000 of the effective doses [11]. Therefore, citric acid was concluded to be the major antihypertensive component of lemon juice. The lemon flavonoids eriocitrin and hesperidin may contribute to the lowering blood pressure by enhancing the antihypertensive effect of citric acid, albeit weakly [11].

Orally administered citric acid is rapidly absorbed and metabolized in vivo. Blood citric acid concentration in the SHRs was estimated to be 21.6 µg/mL (0.112 mmol/L) under normal conditions, which is higher than 11.9 µg/mL (0.0617 mmol/L) in normotensive Sprague–Dawley rats [35]. At a citric acid dose of 90 mg/kg (0.47 mol/kg), the maximum blood concentration was 40.1 µg/mL (0.209 mmol/L). The significant negative correlation between lemon consumption and the SBP and the positive correlation between lemon consumption and blood citric acid levels [10] may indicate that the citric acid in lemons lowers the higher SBP. The maximum antihypertensive effects of orally administered citric acid and lemon juice were observed after 9 h, with a time lag between the maximum blood concentration and the maximum concentration, suggesting that in addition to the direct vasorelaxation effect of citric acid, citric acid may induce antihypertensive effects by modulating the levels of biological substances involved in regulating blood pressure.

The citric acid and lemon juice solutions showed similar vasorelaxation kinetics at the same citric acid concentration, suggesting that citric acid is the major vasodilator in lemon juice. Its vasorelaxant effect was greatly attenuated by the removal of the endothelium, suggesting that citric acid promotes the production of endothelium-derived vasodilators. Flavonoids have been reported to have vasorelaxant effects by lowering blood pressure, mediated via endothelium-dependent or -independent mechanisms, including the stimulation of nitric oxide (NO) release by the endothelium and prostacyclin-induced inhibition of the activity of K and Ca channels in vascular smooth muscle cells [36,37]. GABA has been reported to have a weak vasorelaxant effect, with a 16% vasorelaxant effect at 30 µmol/L [38]. The eriocitrin, hesperidin, and GABA concentrations were 7.9 × 10^−4^ mmol/L, 4.6 × 10^−4^ mmol/L, and 0.021 mmol/L, respectively, when the citric acid concentration in the chamber was 5.0 mmol/L, indicating that their contribution to vasorelaxation in the lemon juice used in the present study was presumed to be minor. When the same amount of AFR-lemon juice was added, the vasorelaxant effects were much weaker than those observed after lemon juice was added. The citric acid and flavonoid contents were much lower in AFR-lemon juice than in the lemon juice, but the GABA contents were the same. The weak vasorelaxant effect of AFR-lemon juice may be due to the presence of GABA (at a concentration of 20 µmol/L) in the chamber. The notable attenuation of vasorelaxation by AFR-lemon juice indicates that citric acid is the major vasodilator in lemon juice. 

Vasodilation has been reported to be an important blood pressure-lowering factor [39]. The maximum blood concentration of citric acid in the SHRs after oral administration was 0.209 mmol/L, with 4.94% vasorelaxation, as calculated from the relaxation curve of citric acid. The attenuation of the vasorelaxant effects of citric acid and lemon juice solution observed after removing the vascular endothelium suggests that the vascular endothelium is partly involved in the vasorelaxant effects of citric acid. In the vascular endothelium, NO, a vasodilator produced from arginine by NO synthase, migrates to the vascular mesothelium by auto-diffusion, activating guanylate cyclase, increasing the cyclic guanosine monophosphate concentration, activating protein kinase G, and decreasing the intracellular Ca ion concentration, and ultimately, relaxing the vascular smooth muscles and dilating the blood vessels [40]. In other words, the vasorelaxant effects of citric acid may involve the enhancement of NO production via vascular endothelial action.

A vasorelaxant response to acidification of HEPES-buffered Krebs solution in the rat thoracic aorta has been reported previously [30]. Therefore, aortic assays were performed in a chamber acidified with HCl. The pH of the chamber was the same as that of the chamber containing 5.0 mmol/L citric acid. The SHR aorta did not relax but contracted after HCl addition (see Table 2). Vasoconstrictions are presumed to be caused by lower pH (acidic) in the extracellular space because higher pH (alkaline) within the intracellular space generally increases the vascular tone of the aorta [41]. The results indicate that citric acid causes vasodilation.

An Increase in the extracellular KCl concentration causes depolarization by inhibiting K outflow to the extracellular space, releasing voltage-gated Ca channels, and allowing Ca to flow into the cell. In vascular smooth muscle cells, Ca-calmodulin complexes generated by elevated intracellular Ca ion concentrations bind to and activate myosin trans-chain kinase, resulting in phosphorylation and enhanced interactions with actin, causing vasoconstriction [42]. Therefore, the inhibition of Ca influx into the vascular smooth muscle cells of KCl-contracted vessels causes vasodilation. Citric acid is expected to inhibit Ca influx into cells by chelating Ca ions. However, the vasorelaxant effects of citric acid at a concentration of 5.0 mmol/mL were weak, and the suppression of Ca ion influx into vascular smooth muscle cells by citric acid-mediated Ca ion chelation was estimated to be limited. Citric acid is thought to induce vasodilation via a mechanism other than the inhibition of Ca ion influx.

The present study demonstrates the antihypertensive and vasodilatory effects of citric acid and lemon juice in SHRs. However, the mechanisms underlying the blood pressure-lowering and vasodilatory activities of citric acid, which are responsible for its antihypertensive effects, are still unknown; further research is required to elucidate these mechanisms.

## 5. Conclusions

The major antihypertensive component of lemon juice in the SHRs was citric acid. The contribution of antihypertensive components other than citric acid, i.e., K, vitamin C, flavonoids (eriocitrin and hesperidin), and GABA, to the blood-pressure-lowering effect of lemon juice, was considered insufficient owing to their low contents. Citric acid mainly acts on the endothelium of the aorta in SHRs and causes vasodilation. Orally administered citric acid is absorbed rapidly into the body, reaching a maximum concentration of 40.1 μg/mL (0.21 mmol/L), indicating the expected vasorelaxant effect of citric acid in vivo. This report indicates that common citric acid may ameliorate blood pressure elevation. However, the mechanism and range of the applications of citric acid remain unclear, and further research is required to validate its effects.

## 6. Patents

The vasodilating action of citric acid has been published by the Japan Patent Office as a pending patent (P2020-186205A, Title of the invention: vasodilator, Inventors: Kozo Nakamura and Masanori Hiramitsu, Applicants: Shinshu University and POKKA SAPPORO Food & Beverage Ltd.).

## Figures and Tables

**Table 1:** Compositions of lemon-derived samples (mg/100 g).

	Citric Acid	Potassium	Vitamin C	Eriocitrin	Hesperidin	GABA
Lemon juice	4506	164	38	2.2	1.3	9.8
AFR-lemon juice	612	117	19	0.034	0.024	9.6

**Table 2:** Vasorelaxation effects caused by citric acid (*n* = 6), lemon juice (*n* = 6), and HCl (*n* = 6, each) at the same pH in the thoracic aortae from SHRs; * vasorelaxations of citric acid and lemon juice are reproduced from Figure 1; HCl: hydrochloric acid.

	Citric Acid	HCl	Lemon Juice	HCl
pH	6.08		6.53	
Concentration (mmol/L)	5.00	8.43 × 10^−4^	5.00	3.01 × 10^−4^
Vasorelaxation (%) *	74.1 ± 3.97	−8.73 ± 7.26	77.7 ± 3.31	−13.2 ± 9.40

## Data Availability

The data that support the findings of this study are available from the corresponding author, K.N., upon reasonable request.
